# FIGO staging for gestational trophoblastic neoplasia: 2026

**DOI:** 10.1002/ijgo.71229

**Published:** 2026-07-19

**Authors:** Hextan Y. S. Ngan, Michael J. Seckl, Neil Horowitz, Annie N. Y. Cheung, Antonio Braga, Paradan K. Sekharan, Susanne Fridsten, Andrea Garrett, Nozomu Yanaihara, Sarikapan Wilailak, Barbara Schmalfeldt

**Affiliations:** ^1^ Department of Obstetrics and Gynecology The University of Hong Kong, Queen Mary Hospital Hong Kong China; ^2^ Department of Medical Oncology, Charing Cross Trophoblastic Disease Center Charing Cross Campus of Imperial College London London UK; ^3^ Department of Obstetrics and Gynecology, Division of Gynecologic Oncology Brigham and Women's Hospital, Dana‐Farber Cancer Institute, Harvard Medical School Boston Massachusetts USA; ^4^ Department of Pathology The University of Hong Kong, Queen Mary Hospital Hong Kong China; ^5^ Department of Obstetrics and Gynecology Rio de Janeiro Federal University. Federal University of the State of Rio de Janeiro. Vassouras University Rio de Janeiro Brazil; ^6^ Department of Obstetrics and Gynecology Institute of Maternal and Child Health, Medical College Calicut India; ^7^ Department of Molecular Medicine and Surgery Karolinska Institutet Stockholm Sweden; ^8^ Department of Nuclear Medicine and Medical Physics Karolinska University Hospital Stockholm Sweden; ^9^ Department of Gynecologic Oncology, Women's and Newborn Services Queensland Trophoblast Centre Royal Brisbane and Women's Hospital Brisbane Queensland Australia; ^10^ Department of Obstetrics and Gynecology The Jikei University School of Medicine Tokyo Japan; ^11^ Department of Obstetrics and Gynecology Faculty of Medicine Ramathibodi Hospital, Mahidol University Bangkok Thailand; ^12^ Clinic and Polyclinic for Gynecology, University Medical Center Hamburg‐Eppendorf Germany

**Keywords:** choriocarcinoma, epithelioid trophoblastic tumor, FIGO cancer report, gestational trophoblastic disease, gestational trophoblastic neoplasia, hydatidiform mole, placental site trophoblastic tumor

## Abstract

Gestational trophoblastic neoplasia (GTN) encompasses a wide range of diseases, which include choriocarcinoma, placental site trophoblastic tumor, epithelioid trophoblastic tumor, and post‐molar persistent or rising levels of human chorionic gonadotrophin. Standardization of the diagnostic criteria and investigative approaches for this uncommon, potentially lethal, yet highly treatable disease is important. Adoption of an evidence‐based staging and scoring system is essential to guide patient management and enable comparison of treatment outcomes. An updated staging system for GTN has been proposed and adopted by the Committee on Women's Cancer of the International Federation of Gynecology and Obstetrics (FIGO) for this purpose. This article highlights the changes introduced in the 2026 system and provide explanatory notes to facilitate interpretation.

## 1 INTRODUCTION

Gestational trophoblastic disease (GTD) comprises a group of disorders arising from various trophoblastic cell types of the placenta and includes lesions histopathologically classified as complete hydatidiform mole, partial hydatidiform mole, invasive and metastatic moles, choriocarcinoma, placental site trophoblastic tumor (PSTT), and epithelioid trophoblastic tumor (ETT).^1^

Molar pregnancy requires close post‐molar monitoring of serum human chorionic gonadotrophin (hCG). Approximately 10%–20% of patients with complete hydatidiform mole have persistently elevated or rising hCG levels after uterine evacuation and are ultimately diagnosed with gestational trophoblastic neoplasia (GTN), with a risk of progression to aggressive choriocarcinoma. Post‐molar GTN has a high cure rate, which partly depends on appropriate staging. The ultimate goal of staging is therefore to distinguish patients who are likely to respond to less intensive chemotherapeutic protocols from those who need more aggressive treatment to achieve remission.

At the International Federation of Gynecology and Obstetrics (FIGO) 2000 meeting, the term gestational trophoblastic neoplasia (GTN) was recommended to replace various terms used to describe the post‐molar spectrum, in addition to choriocarcinoma, PSTT, and ETT. FIGO had already included non‐surgical and non‐pathologic prognostic risk factors, such as serum hCG levels, into the traditional anatomical staging system in 1991.^2^, ^3^, ^4^, ^5^

Although non‐neoplastic trophoblastic lesions, including abnormal (non‐molar) villous lesions, exaggerated placental site reaction, placental site nodule, and atypical placental site nodule (APSN), are not part of the staging of GTN, they represent important differential diagnoses for hydatidiform mole and GTN. Furthermore, while they are not malignant, it has been reported that 10%–15% of APSNs may coexist with or progress to PSTT/ETT.^6^

The Committee on Women's Cancer under FIGO, led by Sarikapan Wilailak, formed a Subcommittee on GTN, chaired by Hextan Y. S. Ngan to evaluate the need for revision of the FIGO staging for GTN, which has been in use since 2000. Members of the subcommittee (Table 1) include gynecological oncologists, medical oncologists, general gynecologists, pathologists, and radiologists from different continents.

**TABLE 1:** Member of the revision on GTN staging and classification subcommittee of FIGO Women Cancer Committee 2024–2026.

Sarikapan Wilailak	Chair FIGO Women's Cancer Committee 2024–2026	Thailand
Hextan YS Ngan	Chair of FIGO GTN staging subcommittee	Hong Kong
Andrea Garrett	Gynecological Oncologist	Australia
Neil Horowitz	Gynecological Oncologist	USA
Antonio Braga	Gynecological Oncologist	Brazil
P. K. Sekharan	Gynecologist	India
Nozomu Yanaihara	Gynecological Oncologist, Liaison Person FIGO	Japan
Michael Seckl	Medical Oncologist	UK
Susanne Fridsten	Nuclear Medicine Radiologist	Sweden
Annie N. Y. Cheung	Pathologist	Hong Kong
Barbara Schmalfeldt	Chair FIGO Women's Cancer Committee 2026–2028	Germany

Abbreviation: GTN, gestational trophoblastic neoplasia.

After discussion during several meetings, a draft proposal was submitted to the Committee on Women's Cancer for comment before feedback was sought from various stakeholders, including the American Joint Committee on Cancer (AJCC), Asian Society of Gynecologic Oncology (ASGO), Australian Society of Gynecologic Oncology (ASGO), American Society for Radiation Oncology (ASTRO), Chinese Society of Gynecologic Oncology (CSGO), European Society of Gynecological Oncology (ESGO), European Society of Pathology (ESP), European Society for Radiotherapy and Oncology (ESTRO), European Society of Urogenital Radiology (ESUR), Gynecologic Cancer Intergroup (GCIG), International Collaboration on Cancer Reporting (ICCR), International Gynecologic Cancer Society (IGCS), International Society of Gynecological Pathologists (ISGyP), International Society for the Study of Trophoblastic Diseases (ISSTD), National Comprehensive Cancer Network (NCCN), Society of Gynecologic Oncology (SGO), and Union for International Cancer Control (UICC). Responses were received from two thirds of the stakeholders.

After compiling the feedback, an updated proposal was prepared and presented to the Committee on Women's Cancer and finally at the FIGO Congress 2025 in Cape Town, South Africa, where the final proposal was adopted. Here, we present the revised 2026 FIGO GTN staging and summarize the changes and the rationale behind them.

## 2 COMMENTS ON GTN STAGING 2000

Over the years, the Committee has received feedback with various suggestions, misconceptions or misinterpretations of the staging and risk‐scoring tables. In this revision, in addition to updating the staging and scoring systems themselves, a substantial number of explanatory notes were added to address previously identified areas of difficulty.

Requests were also made for changes to the stage and risk classifications. Some of these were adopted, whereas others were deferred because of insufficient supporting evidence and the need for further data collection.

To facilitate accurate staging and risk‐score stratification, it was considered essential to standardize diagnostic criteria and investigative methods. Several amendments were therefore introduced and supplemented with explanatory notes for ease of reference. As technological advances, particularly in imaging, continue to change rapidly, further amendments may be incorporated through future updates to clinical practice guidelines.^7^

## 3 CRITERIA FOR DIAGNOSIS OF GTN 2026

For clarity, the criterion for a rise in hCG was defined as “an increase in hCG of over 10% across three consecutive weekly measurements over a period of at least 2 weeks (i.e. days 1, 7, 14).” “A plateau in hCG, defined as a change of less than 10% (rise or fall) across four measurements over a period of at least 3 weeks (i.e. days 1, 7, 14, 21)” also satisfies the definition of GTN. The diagnostic criteria based on histologic GTN types were expanded to specify the qualifying pathologies, with “gestational choriocarcinoma, PSTT, and ETT” added (Table 2).

**TABLE 2:** Criteria for the diagnosis of GTN 2026.

GTN may be diagnosed when the plateau of hCG (< 10% rise or fall) lasts for four measurements over a period of 3 weeks or longer (i.e. days 1, 7, 14, 21)
GTN may be diagnosed when there is a rise of > 10% of hCG of three weekly consecutive measurements or longer, over at least a period of 2 weeks or more (i.e. days 1, 7, 14)
GTN is diagnosed if there is a histopathological diagnosis of gestational choriocarcinoma, PSTT, or ETT

Abbreviations: ETT, epithelioid trophoblastic tumor; GTN, gestational trophoblastic neoplasia; hCG, human chorionic gonadotropin; PSTT, placental site trophoblastic tumor.

The diagnostic criterion stating that “the hCG level remains elevated for 6 months or more” was removed, as previously noted in the FIGO report.

## 4 IMAGING INVESTIGATIONS 2026

The choice of imaging methods depends on risk and availability (Table 3).

**TABLE 3:** Imaging investigations of GTN 2026.

Pelvis—USS with Doppler or MRI depending on risk and availability
Abdomen—CT, MRI, or USS depending on risk and availability
Chest CT and CXR are appropriate for the diagnosis of lung metastases and for counting the number of lung metastases to evaluate the risk score. Only lesions ≥ 1 cm should be counted
If lung metastases are ≥ 1 cm or choriocarcinoma, contrast‐enhanced MRI of the brain plus CT or contrast‐enhanced MRI of the abdomen/pelvis are required to assess further potential metastatic disease sites
Since GTN can double in volume every 24–48 h in some patients, imaging should be carried out as close as possible to the start of chemotherapy or surgical intervention

Abbreviations: CT, computed tomography; CXR, chest radiograph; GTN, gestational trophoblastic neoplasia; MRI, magnetic resonance imaging; USS, ultrasound.

### 4.1 Low‐risk disease

For suspected low‐risk disease, chest radiography (CXR) and pelvic ultrasonography (USS) with Doppler or magnetic resonance imaging (MRI) are recommended.

For assessment of tumor size within the uterus, USS with Doppler is typically sufficient to measure the maximal tumor diameter. Uterine volume or overall size is not included in the FIGO scoring system. In addition, the presence of clots should be considered, as these may confound tumor assessment. Pelvic computed tomography (CT) scan is not recommended for tumor size measurement because of its limited soft‐tissue contrast, which may make it difficult to distinguish tumor tissue from blood clots or the uterine wall.

MRI can be used to assess pelvic disease and although it provides the highest accuracy, it is usually not required in patients with post‐molar low‐risk disease.

Chest CT is indicated if lung metastases are identified or suspected on CXR. Only lesions greater than 1 cm are included in the scoring system. If lung metastases measuring 1 cm or more are detected on CXR or chest CT, brain MRI together with abdominal CT or MRI is recommended to assess for additional metastatic sites. Pulmonary micrometastases have not been shown to affect treatment outcomes and therefore should not be used in determining stage or risk score.^8^

Positron emission tomography (PET)‐CT should not be performed routinely but may be considered in patients with high‐risk GTN.^9^ In cases with very high hCG concentrations (e.g. > 400 000 mIU/mL), the risk of metastases at other sites is increased, even in the absence of metastases on CXR, and additional imaging may be considered.

### 4.2 High‐risk disease

For suspected high‐risk disease and in any patient diagnosed with choriocarcinoma, more extensive investigations are required, including contrast‐enhanced MRI of the brain and pelvis, together with CT of the chest and abdomen.

For abdominal assessment, USS may have a role in low‐resource settings; however, it is insufficient for evaluation of liver metastasis for staging purposes. Contrast‐enhanced CT should be the first‐line modality for detection of liver metastases, with MRI (preferably with intravenous contrast) reserved as a problem‐solving tool for cases in which CT contrast is contraindicated.

MRI is the preferred imaging modality for assessment of the brain. CT of the brain should be reserved for acute situations when MRI is not immediately available.

### 4.3 PSTT/ETT

For staging of PSTT/ETT, contrast‐enhanced pelvic MRI should be mandatory for accurate assessment of myometrial and parametrial involvement, given the tendency of these tumors toward local invasion rather than widespread metastasis. Ultrasound and CT are insufficient for local staging.

### 4.4 Timing of investigation

Since GTN can double in volume within 24–48 h in some patients, investigations should be conducted as close as possible to the start of chemotherapy or surgical intervention. We recommend following the investigations outlined in the recent Practical Guidelines for the Treatment of Gestational Trophoblastic Disease, developed through collaboration between the European Organization for the Treatment of Trophoblastic Disease (EOTTD), European Society of Gynecologic Oncology (ESGO), Gynecologic Cancer InterGroup (GCIG), and International Society for the Study of Trophoblastic Diseases (ISSTD).^7^ These guidelines generally undergo revision more frequently than formal staging recommendations, allowing updated guidance to be distributed as new technologies or knowledge become available.

## 5 FIGO ANATOMICAL STAGING FOR GTN 2026

The anatomical stage from I to IV (Table 4) was retained, with additional clarification on:

- Adding cervix under stage I
- Adding broad ligament under stage II
- Adding lymph node metastasis found below the diaphragm under stage III
- Adding lymph node metastasis found above the diaphragm under stage IV

Lymph node status was added because imaging is frequently performed, particularly in patients with high‐risk disease, and often identifies lymph node metastasis. Although the available data are controversial, some studies suggest that lymph node metastasis may be associated with poorer outcomes; however, the impact of lymph node metastases on survival remains unclear. On this basis, the committee considered it important to collect additional data regarding the potential impact of nodal involvement in patients with GTN. The proposal is to include lymph node staging, as used in other solid tumors, by classifying them within stages III and IV and limited to lymph nodes measuring 1 cm or greater in short‐axis diameter.^10^

**TABLE 4:** FIGO anatomical staging for GTN 2026.

FIGO stage	Description
I	Gestational trophoblastic tumors strictly confined to the uterine corpus and cervix
II	Gestational trophoblastic tumors extending to the adnexa or to the vagina or broad ligaments, but limited to the genital structures
III	Gestational trophoblastic tumors extending to the lungs, with or without genital tract involvement, lymph node metastasis found below the diaphragm^a^
IV	All other metastatic sites including lymph node metastasis above the diaphragm^a^

Abbreviation: GTN, gestational trophoblastic neoplasia.

^a^ For lymph node metastasis, the notation “r” (imaging) or “p” (pathology) should be added to indicate the method used to allocate the case to stage III or IV. For example, lymph node metastasis identified on imaging below the diaphragm should be classified as stage III LN r. A lymph node short‐axis diameter ≥ 10 mm should be used to define metastasis and for counting the number of assessable likely involved lymph nodes on imaging.

However, it is important to note that surgical staging of lymph node status is not recommended, as there are currently insufficient data to support the view that such an approach would influence additional treatment strategies or improve survival outcomes. Furthermore, GTN spreads mainly via the hematogenous route.

Similar to staging in cervical cancer, lymph node metastases should include the notation “r” (imaging) or “p” (pathology) to indicate the basis on which findings are used to allocate the case to stage III or IV. For example, lymph node metastasis identified on imaging below the diaphragm should be designated as stage III LN r.

The imaging modality or pathological technique used should always be documented.

A lymph node short‐axis diameter smaller than 10 mm on imaging is suggested as the threshold for defining assessable likely involved lymph nodes.

## 6 FIGO‐MODIFIED WHO RISK SCORE ON PROGNOSTIC FACTORS 2026

### 6.1 Highlight on risk scores (Table 5)

**TABLE 5:** FIGO‐modified WHO risk score 2026.

Prognostic factor	Risk score			
	0	1	2	4
Age^a^ (years)	< 40	≥ 40		
Antecedent pregnancy^b^	Hydatidiform mole	Abortion/miscarriage/ectopic	Term pregnancy	
Interval months from index pregnancy^b^	< 4	4–6	7–12	> 12
Pretreatment hCG^c^ (mIU/mL)	< 1000	1000 to < 10 000	10 000 to < 100 000	≥ 100 000
Largest size of tumor^d^ (in uterus/metastatic site) (cm)	< 3	3–5	> 5	
Site of metastases^e^	Lung, vagina	Spleen, kidney	Gastrointestinal tract	Brain, liver
Number of metastases identified^f^	–	1–4	5–8	> 8
Total score:				

Abbreviation: GTN, gestational trophoblastic neoplasia.

^a^ Age refers to the age of the patient at diagnosis of GTN.

^b^ If there has been more than one previous pregnancy, the most recent pregnancy, including the current pregnancy, should be assigned as the index antecedent pregnancy.

^c^ hCG level used should be the value measured at the diagnosis of GTN and before initiation of treatment, whether surgical (after hysterectomy/lobectomy) or medical. The hCG level measured before evacuation of a molar pregnancy, which is not GTN, should not be used as the reference value or for calculation of the risk score. As GTN can double in volume every 24–48 h in some patients, serum hCG measurements should be carried out as close as possible to the start of chemotherapy or surgical intervention.

^d^ Assessment of tumor size should include both uterine and metastatic lesions. Measurement should relate to tumor size rather than uterine size. As GTN can double in volume every 24–48 h in some patients, imaging should be carried out as close as possible to the start of chemotherapy or surgical intervention.

^e^ The uterus is not considered a metastatic site, whereas ectopic choriocarcinoma should be considered a primary site.

^f^ Uterine tumors should not be included when counting the number of metastases. Vaginal metastases should be assessed both clinically and by imaging. Lung metastases measuring ≥ 1 cm can be counted using either chest CT or chest radiography.

- Use of a scoring sheet is recommended, with the relevant prognostic factors circled under each score category, the total score calculated, and the completed sheet kept in a medical record.
- Abortion/miscarriages should be included, encompassing both spontaneous and induced pregnancy losses, as well as ectopic pregnancies occurring in tubal, ovarian, or other extrauterine sites.^11^
- The risk score should not be applied to management of PSTT or ETT.
- It should be clarified that assessment relates to tumor size rather than uterine size.
- The listed metastatic sites were not updated and continues to include the vagina, lung, spleen, kidneys, gastrointestinal tract, liver, and brain, with no new anatomical locations added. This is because a fully comprehensive list of metastatic sites is not feasible. Clinical judgment is needed for sites other than those listed. Moreover, metastases to organs outside the current list should be regarded as high‐risk disease.
- Regarding the number of metastases, vaginal metastasis may be identified by clinical examination or imaging. Lung metastasis may be assessed using CXR or chest CT. Other sites should be evaluated using CT and/or MRI, as described above.
- The prognostic factor relating to previous chemotherapy use in the earlier scoring system has been removed from the current version. As restaging is not recommended in cases of resistance, recurrence, or relapse, and prophylactic chemotherapy is not regarded as prior chemotherapy, this factor was considered no longer applicable.
- In summary, seven prognostic factors considered clinically significant remain within the scoring table. As there are no robust data supporting changes to the weighting of these factors, the scoring format remains unchanged.^12^, ^13^

### 6.2 More explanatory notes on risk scores based on prognostic factors

- Age: Age refers to the patient's age at the time of GTN diagnosis.^14^
- Antecedent pregnancy and interval from pregnancy: If there has been more than one previous pregnancy, the most recent pregnancy, including the current pregnancy, should be designated as the index antecedent pregnancy. Where resources permit, genetic testing such as short tandem repeat (STR) genotyping can be used to confirm the index pregnancy in cases with multiple previous pregnancies.^15^, ^16^, ^17^
- Pretreatment hCG level: The hCG level used should be the value measured at the time of GTN diagnosis and before initiation of treatment, whether surgical (e.g. after hysterectomy/lobectomy) or medical. The hCG level recorded before evacuation of a molar pregnancy, which itself does not constitute GTN, should not be used as the reference value. Because GTN may double in volume within 24–48 h in some patients, serum hCG measurement should be performed as close as possible to the start of chemotherapy or surgical intervention.
- Largest tumor size: Assessment of tumor size includes both uterine and metastatic lesions. The tumor size should be measured, rather than the uterus size. Care should be taken to differentiate tumor tissue from blood clots. Tumor size can be assessed using MRI, USS with Doppler, or pathology, whereas CT should not be used. As GTN can double in volume within 24–48 h in some patients, imaging should be performed as close as possible to the initiation of chemotherapy or surgical intervention
- Site of metastasis: The uterus is not considered a metastatic site, whereas ectopic choriocarcinoma should be regarded as a primary tumor site. Metastatic sites not included in the standard list of the scoring table are typically associated with multiple metastatic lesions and therefore would not increase the score.
- Number of metastases: The uterine tumor should not be included when counting metastatic lesions. Vaginal metastases should be assessed both clinically and by imaging for inclusion in the metastatic count. Lung metastasis measuring 1 cm or greater can be counted using either chest CT or CXR.

## 7 RISK CLASSIFICATION BASED ON RISK SCORE 2026

### 7.1 Low‐risk group

Low‐risk disease is defined as risk scores of 0 to 6. Scores of 5 and 6 in the low‐risk group are recognized as being associated with higher rates of chemoresistance (Table 6). Nevertheless, introducing an additional intermediate‐risk group or altering the current cutoff value is not recommended at present.

**TABLE 6:** FIGO GTN risk classification 2026.

Risk groups
0–6: Low risk
7–12: High risk
≥ 13: Ultra‐high risk

Patients with scores of 5 or 6 combined with additional risk factors may benefit from multi‐agent chemotherapy as first‐line treatment. Such risk factors include:

1. Post‐molar GTN without metastasis but with a pretreatment hCG level of 410 000 IU/L or above.
2. Either post‐molar GTN with metastasis or choriocarcinoma without metastasis and a pretreatment hCG of 150 000 IU/L or above.
3. Choriocarcinoma with metastasis, with any elevated hCG level.^7^, ^18^

### 7.2 High‐risk group

High‐risk disease is defined as a risk score from 7 to 12. Documentation and notation of stage and score remain unchanged, i.e. stage: score.

### 7.3 Ultra‐high‐risk group

A new category has been introduced for ultra‐high‐risk disease, defined as a risk score of 13 or above. Patients in this group have a poorer prognosis, and the use of induction chemotherapy has been shown to reduce mortality.^19^

## 8 GESTATIONAL CHORIOCARCINOMA

There is no need for a separate staging and scoring system for choriocarcinoma, as it can be assessed using the current GTN staging and scoring system.

However, imaging for patients diagnosed with choriocarcinoma needs to be more extensive from the outset and should not be limited to pelvis USS with Doppler and CXR. Instead, contrast‐enhanced CT of the chest and abdomen as well as contrast‐enhanced MRI of the brain and pelvis should be performed when available.^7^, ^17^, ^20^

Where indicated and available, genetic testing such as STR genotyping can be used to distinguish gestational from non‐gestational choriocarcinoma. In addition, genotyping may help identify the index pregnancy for evaluation of the antecedent pregnancy interval.^15^, ^16^ Pathologists are advised to follow the reporting dataset recommended by the ICCR.^21^

## 9 PSTT/ETT

A new FIGO staging system for PSTT and ETT has been introduced (Table 7).

**TABLE 7:** FIGO staging for PSTT/ETT 2026.

FIGO stage	Description
I	Gestational trophoblastic tumors strictly confined to the uterine corpus and cervix With good prognostic factor: < 48 months interval from previous pregnancy^a^ With poor prognostic factor: ≥ 48 months interval from previous pregnancy^a^
II	Gestational trophoblastic tumors extending to the adnexa or to the vagina or broad ligaments, but limited to the genital structures With good prognostic factor: < 48 months interval from previous pregnancy^a^ With poor prognostic factor: ≥ 48 months interval from previous pregnancy^a^
III	Gestational trophoblastic tumors extending to the lungs, with or without genital tract involvement; lymph node metastases found below the diaphragm^b^ With good prognostic factor: < 48 months interval from previous pregnancy^a^ With poor prognostic factor: ≥ 48 months interval from previous pregnancy^a^
IV	All other metastatic sites, including lymph node metastases above the diaphragm^b^

^a^ State the type of pregnancy.

^b^ For lymph node metastasis, the notation “r” (imaging) or “p” (pathology) should be added to indicate the method used to allocate the case to stage III or IV. For example, lymph node metastasis identified on imaging below the diaphragm should be classified as stage III LN r. A lymph node short‐axis diameter ≥ 10 mm should be used to define metastasis and for counting the number of assessable likely involved lymph nodes on imaging.

PSTT and ETT exhibit behaviors different from other forms of GTN. Staging should therefore be based on anatomical and clinical prognostic factors. Two factors in particular—stage IV and an antecedent pregnancy interval of 48 months or longer—are important prognostic factors.^22^, ^23^, ^24^

It is recommended to stage using the same anatomical staging as GTN, but not including the modified WHO score. Instead, for stages I to III, PSTT/ETT treated ≥ 48 months interval from previous pregnancy, is considered as having a poor prognostic factor (PP) whereas treatment started < 48 months is considered as having a good prognostic factor (GP). All stage IV cases have poor prognosis.

Notation of the stage and prognostic factor is similar to that of GTN, i.e., stage:GP or PP for stage I to III. Where indicated and available, genetic testing such as STR genotyping can be used to identify the index pregnancy for evaluation of the antecedent pregnancy interval.^15^, ^16^ If genetic testing is unavailable and more than one pregnancy preceded the diagnosis of PSTT or ETT, the most recent pregnancy, including the current pregnancy, should be considered the index antecedent pregnancy.

Although there are limited data on lymph node metastasis, as in other forms of GTN, lymph node metastasis impacts poorer outcomes in cases of PSTT or ETT. Lymph node metastases below the diaphragm are associated with a better prognosis than nodal metastases above the diaphragm. As a result, patients with lymph node involvement below and above the diaphragm are classified as stage III and IV, respectively, in keeping with staging in other gynecological cancers.^10^ However, given the limited and controversial evidence, surgical lymph node staging is not recommended, and further data collection is required.

For lymph node metastasis, the notation “r” (imaging) or “p” (pathology) should be added to indicate the basis of findings used for stage allocation. For example, lymph node metastasis identified on imaging below the diaphragm should be designated as stage III LN r. A lymph node short‐axis diameter of 10 mm or greater is suggested as the threshold for identifying lymph nodes likely to harbor metastatic disease.

Pathologists are advised to follow the reporting dataset recommended by the ICCR.^21^

## 10 SUMMARY

- The revisions mainly aim to clarify and better define the diagnostic criteria, as well as to incorporate the role of imaging and pathology. To better use the staging and scoring system, this article includes more detailed explanatory notes.
- Minor changes have been made to the anatomical staging and scoring system, including removal of the history of previous chemotherapy.
- A new ultra‐high‐risk category has been added, with treatment implications, as induction chemotherapy in this group has been associated with better outcomes.
- There is a new staging system for PSTT/ETT based mainly on anatomical staging with the addition of one clinical prognostic factor, interval from antecedent pregnancy for stage I to III. Scoring is no longer used.
- Because GTN is an uncommon gynecological malignancy, it is important that consistent diagnostic criteria and interpretation of investigations are used to standardize definitions and GTN score evaluation. Equally important is adoption of a uniform staging and scoring system for generation of more robust data using the same reference for comparison in research to arrive at more solid conclusions that can benefit our patients.
- The new revised staging and scoring system is a milestone guiding us to further improvements. Clinicians treating GTN should adopt these new recommendations moving forward.

## AUTHOR CONTRIBUTIONS

All authors have met the following four criteria: substantial contributions to the conception or design of the work; or the acquisition, analysis, or interpretation of data for the work; drafting the work or reviewing it critically for important intellectual content; final approval of the version to be published; and agreement to be accountable for all aspects of the work in ensuring that questions related to the accuracy or integrity of any part of the work are appropriately investigated and resolved.

## CONFLICT OF INTEREST STATEMENT

The authors have no conflicts of interest.

## Data Availability

Data sharing is not applicable to this article as no new data were created or analyzed in this study.
